# Correction: Cryptococcus neoformans resists to drastic conditions by switching to viable but non-culturable cell phenotype

**DOI:** 10.1371/journal.ppat.1008070

**Published:** 2019-09-17

**Authors:** Benjamin Hommel, Aude Sturny-Leclère, Stevenn Volant, Nathanaël Veluppillai, Magalie Duchateau, Chen-Hsin Yu, Véronique Hourdel, Hugo Varet, Mariette Matondo, John R. Perfect, Arturo Casadevall, Françoise Dromer, Alexandre Alanio

The Data Availability statement for this paper is incorrect. The correct statement is: All RNA sequence data from this study have been submitted to NCBI (https://www.ncbi.nlm.nih.gov/geo) under accession number GSE118549. The mass spectrometry proteomics data have been deposited to the ProteomeXchange Consortium via the PRIDE partner repository with the dataset identifier PXD012570 http://proteomecentral.proteomexchange.org/cgi/GetDataset.

Additionally, the authors inadvertently omit Dr. Caitlin Pepperell in their Acknowledgements section.

The correct Acknowledgements section should read: We would like to acknowledge Christina A. Cuomo, Laurent Châtre, Guilhem Janbon, Jean-Yves Coppée, Pierre Rocheteau, Pierre Henri Commere, Christine Schmitt, Olivier Gorgette, Jacomine Krijnse-Locker, Caroline Proux, Rachel Legendre for their comments on this work and help at different steps of the study.
