# Supplementary material for: Cryptococcus neoformans resists to drastic conditions by switching to viable but non-culturable cell phenotype
Source: PLoS Pathog. 2019 Jul 29;15(7):e1007945. doi: 10.1371/journal.ppat.1007945 (PMC6687208; doi:10.1371/journal.ppat.1007945)
Supplement: S8 Table — (DOCX) [file ppat.1007945.s008.docx]

**Table S8: Strains used in this study**

| Strain denomination | Reference or Source |
| --- | --- |
| H99O | J. Heitman (Duke University, Durham, NC, USA |
| KN99α | Madhani collection |
| *CNAG_02489Δ* KN99α | Madhani collection |
| *CNAG_04688Δ* KN99α | Madhani collection |
| *CNAG_06628Δ* KN99α | Madhani collection |
| *CNAG_03019Δ* KN99α | Madhani collection |
| *CNAG_00490Δ* KN99α | Madhani collection |
| *CNAG_07747Δ* KN99α | Madhani collection |
| *CNAG_04531Δ* KN99α | Madhani collection |
| *CNAG_00524Δ* KN99α | Madhani collection |
| *CNAG_05721Δ* KN99α | Madhani collection |
