# Supplementary figures and images for: Cryptococcus neoformans resists to drastic conditions by switching to viable but non-culturable cell phenotype
Source: PLoS Pathog. 2019 Jul 29;15(7):e1007945. doi: 10.1371/journal.ppat.1007945 (PMC6687208; doi:10.1371/journal.ppat.1007945)

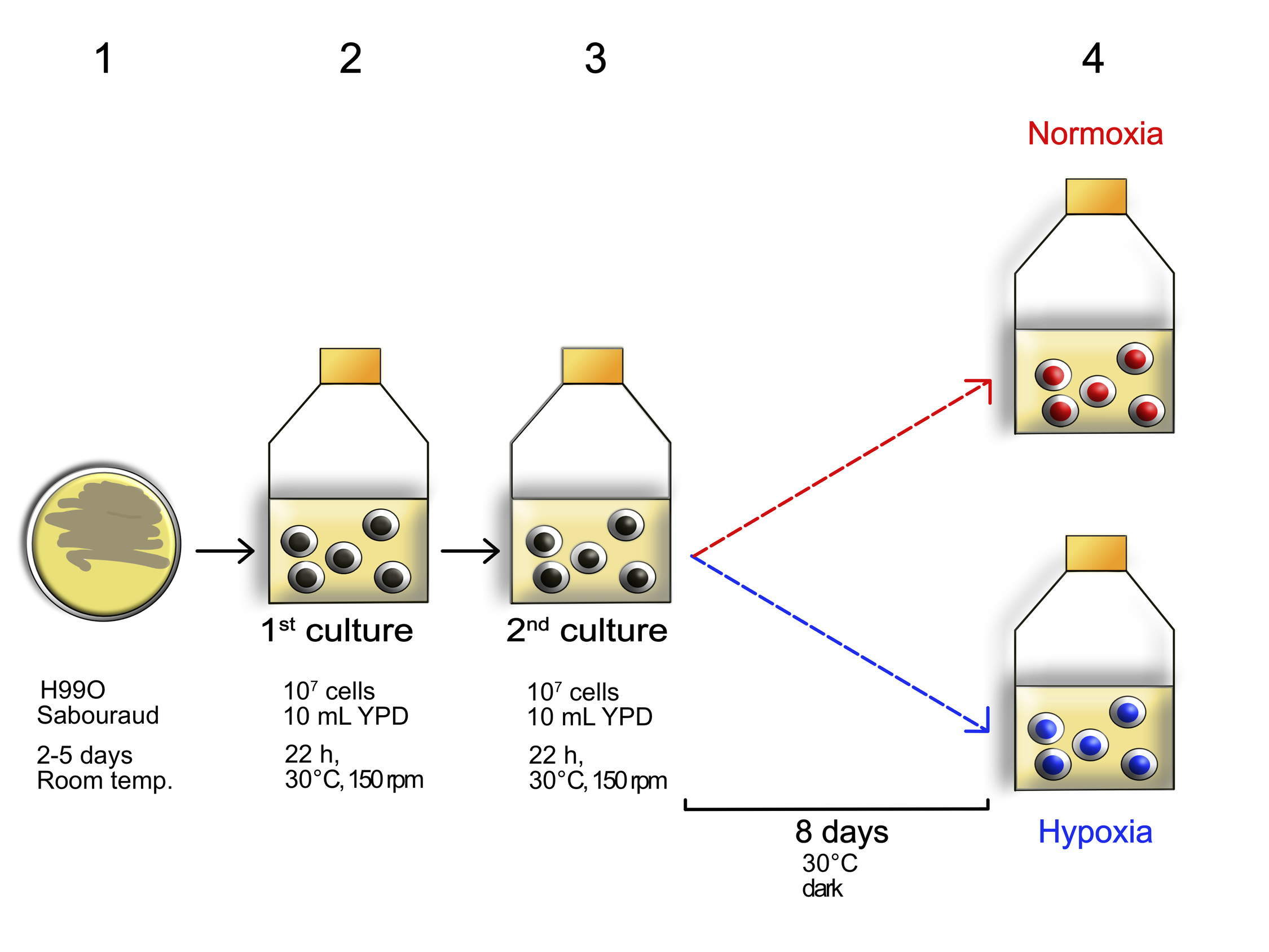

Supplement: S1 Fig — The protocol followed several steps: (1) Cryptocococcus neoformans cells of a stock culture frozen at -80°C were grown on Sabouraud agar plate for 2 to 5 days at room temperature; (2) 107 cells were then suspended in 10 mL yeast extract peptone dextrose 2% (YPD) and incubated with lateral shaking (150 rpm) at 30°C for 22 hours (stationary phase-STAT); (3) 100 μl, 2x10^7^ cells were incubated again until the STAT and then placed in hypoxia or normoxia in the dark at 30°C (4) for up to 8 days when the resultant cells in hypoxia and normoxia were analyzed. (TIF) [file ppat.1007945.s010.tif]

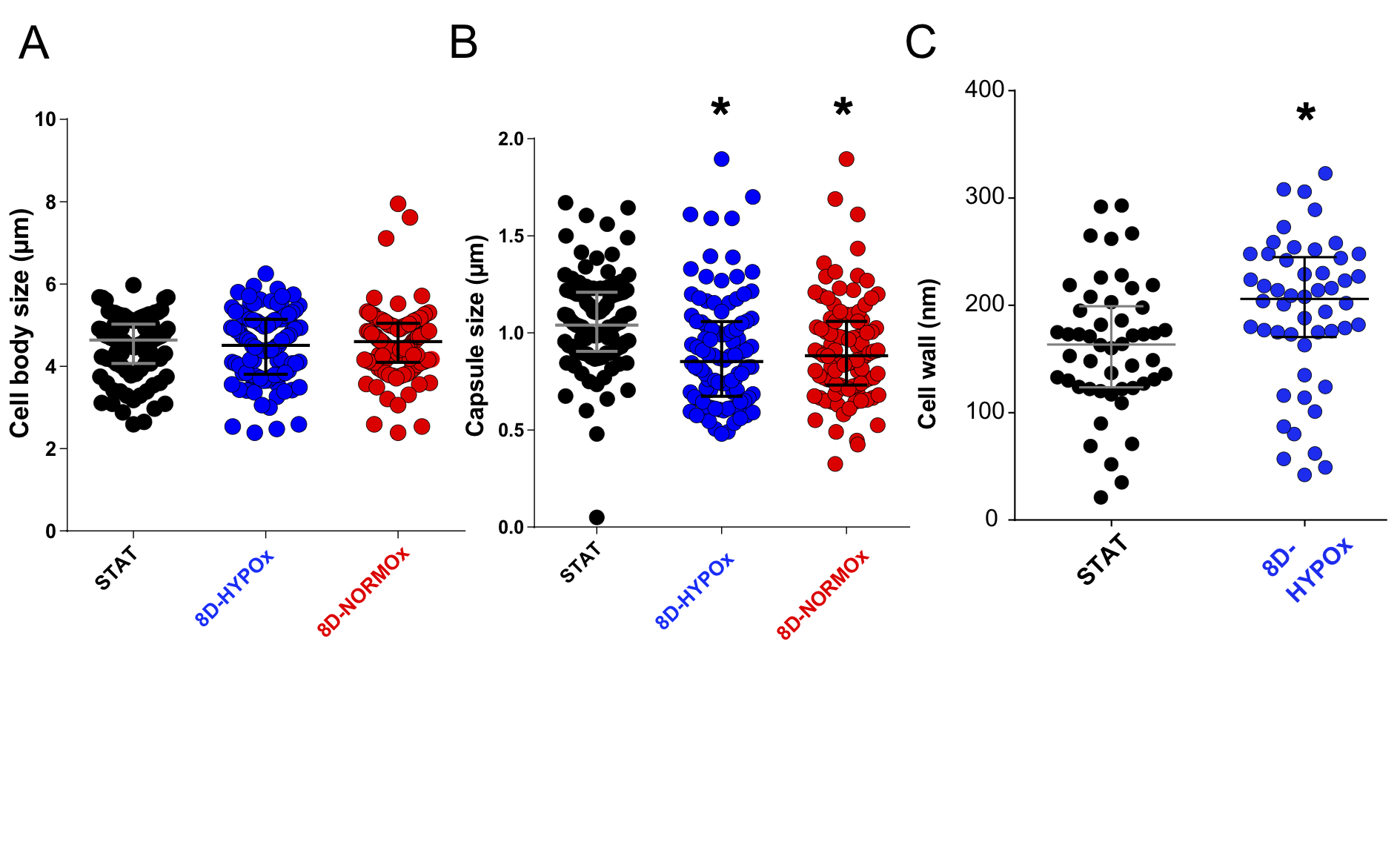

Supplement: S2 Fig — A. Median cell sizes were similar in 8D-HYPOx, 8D-NORMOx and STAT. B. Median capsule size was lower in 8D-HYPOx, 8D-NORMOx compared to STAT (*p<0.01, 100 cells measured). C. Cell wall was thicker in 8D-HYPOx compared to STAT (*p = 0.0152, 50 cells measured). (TIF) [file ppat.1007945.s011.tif]

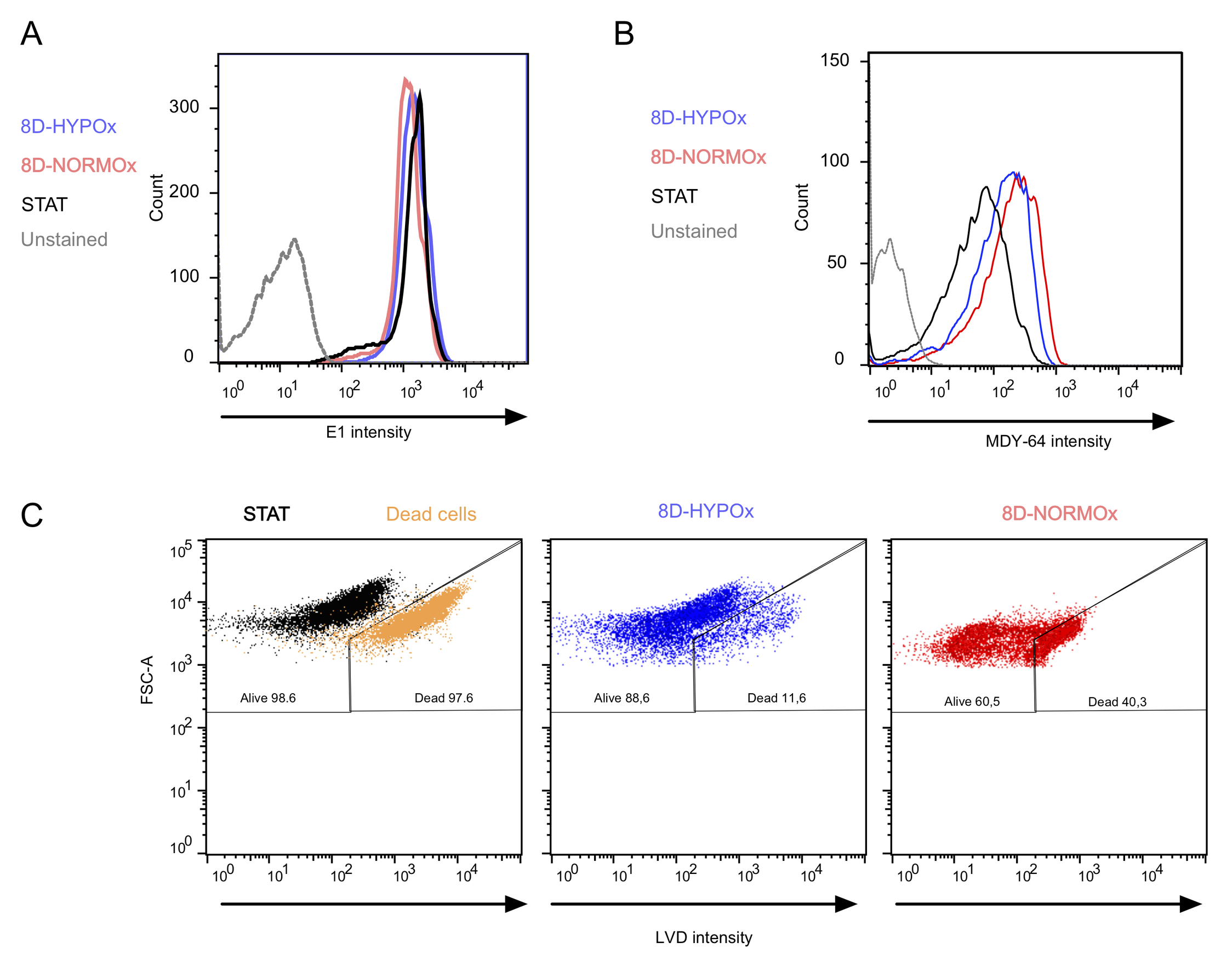

Supplement: S3 Fig — A. Histograms of fluorescence intensity showing no difference in binding pattern after staining with the E1 anti-glucuronoxylomannan monoclonal antibody B. More intense vacuolar staining with MDY-64 in 8D-HYPOx, 8D-NORMOx conditions (one representative experiment out of the 3 independent experiments performed is shown) C. Viability was assessed by membrane permeability staining (LIVE/DEAD, LVD) showing almost 99% of live cells in STAT cells and 100% of dead cells in heat-killed cells (Left panel). Plasma membrane was intact for more (87.2% [83.2–88.6]) cells in 8D-HYPOx, than in 8D-NORMOx (53.8% [50.9–60.5]). Experiments were done in triplicate and a representative diagram is shown. (TIF) [file ppat.1007945.s012.tif]

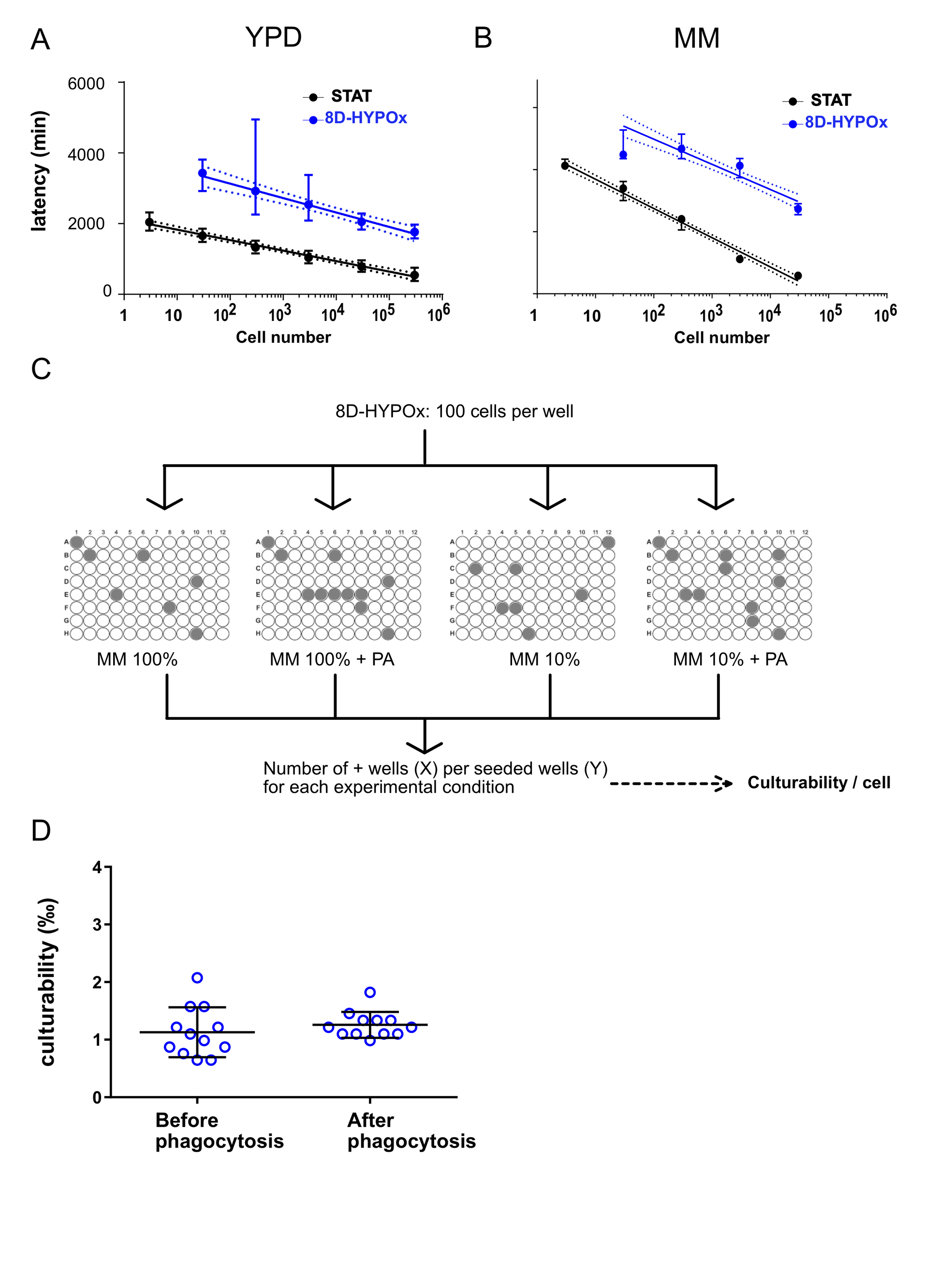

Supplement: S4 Fig — Growth of STAT and 8D-HYPOx cells was assessed using the BioScreen apparatus. Serial dilutions of 8D-HYPOx and STAT cells increased the latency in YPD (A) and in MM (B). Latency curves extrapolations showed for both STAT and 8D-HYPOx cells, global latency was decreased in YPD compared to MM. Each point represents the median ± IQR of the latency of 3 independent experiments. C. Experimental set up used for the determination of the probability of growth per cell (culturability). Hundred yeasts cells per well were plated in 96-well plates in each condition (MM at 10 or 100% ± pantothenic acid (PA) at 125μM). The number of positive wells per plate were determined and the probability of growth per plate was calculated (see M&M section) D. 8D-HYPOx were exposed to macrophages during two hours in the presence of opsonin. Culturability was similar in phagocytosed 8D-HYPOx cells compared to controls. Each dot represents the calculated culturability. Two independents experiments are pooled. (TIF) [file ppat.1007945.s013.tif]

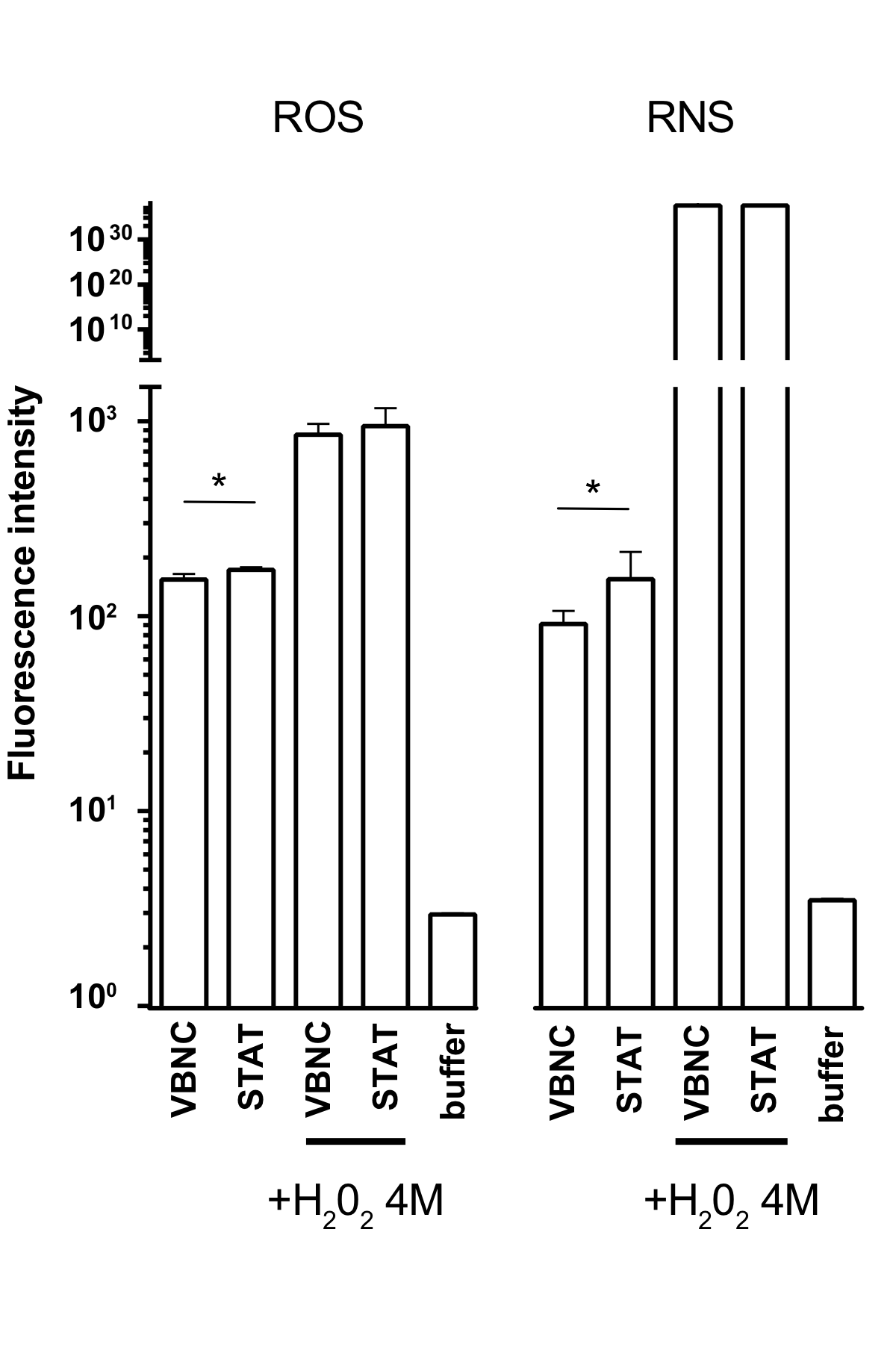

Supplement: S5 Fig — Testing the ROS and RNS in VBNC vs STAT cells found a slight decreased in VBNC. ROS (left panel) and RNS (right panel) productions were measured using fluorescence probes and were significantly lower in VBNC compared to STAT cells respectively (*p<0.01). As expected, the addition of H202 as a positive control increased ROS and RNS levels. (TIF) [file ppat.1007945.s014.tif]

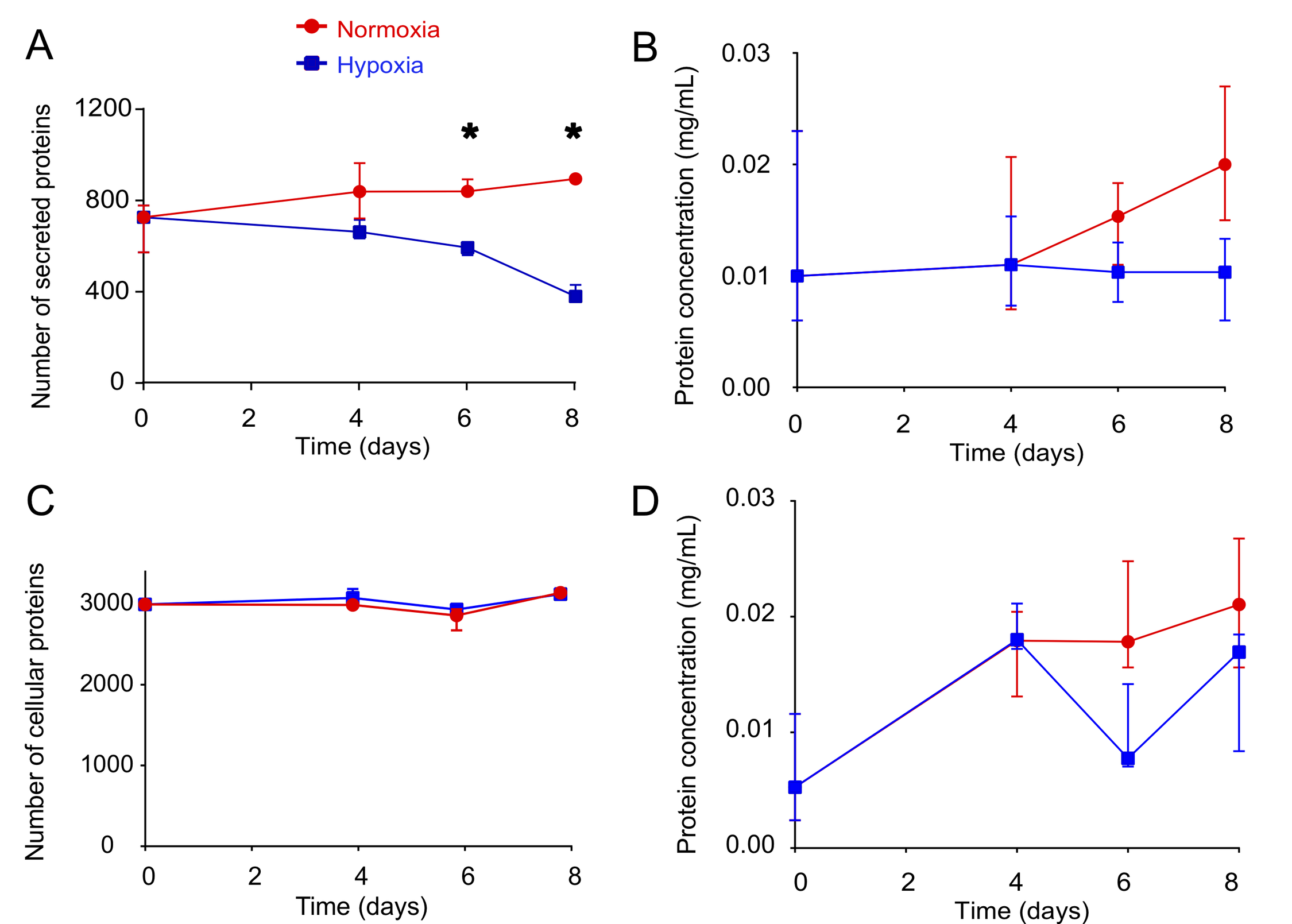

Supplement: S6 Fig — The number of secreted protein number significantly decreased over time in VBNC (* p<0.01) (A) while the protein concentration tended to increase in 8D-NORMOx (B). The number of cellular proteins remained stable in both conditions (C) while the protein concentration tended to increase (D). The experiments were done in triplicates (median±IQR]. (TIF) [file ppat.1007945.s015.tif]

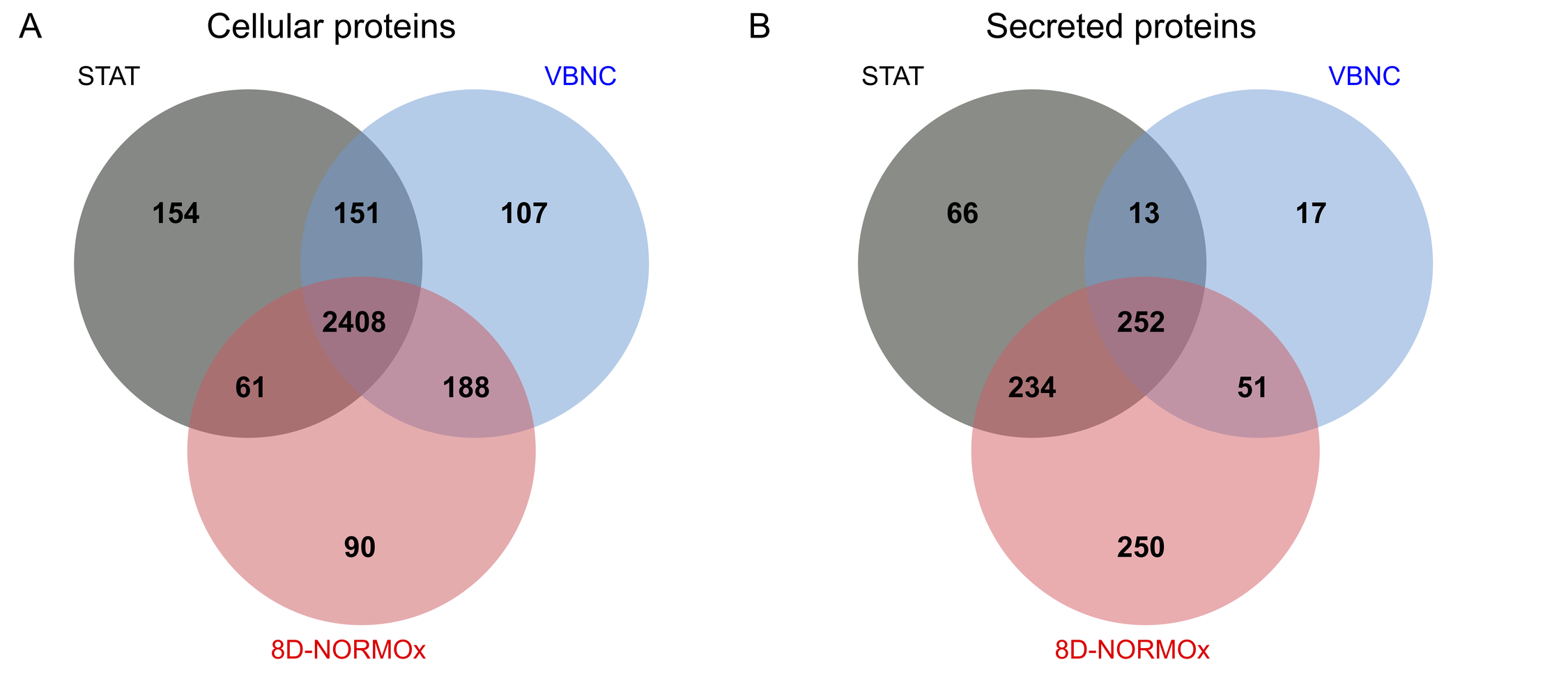

Supplement: S7 Fig — A. A hundred and seven cellular proteins were only present in hypoxia and 2408 were common to the 3 experimental conditions. B. Seventeen secreted proteins were only present in hypoxia and 252 were common to the 3 experimental conditions. (TIF) [file ppat.1007945.s016.tif]

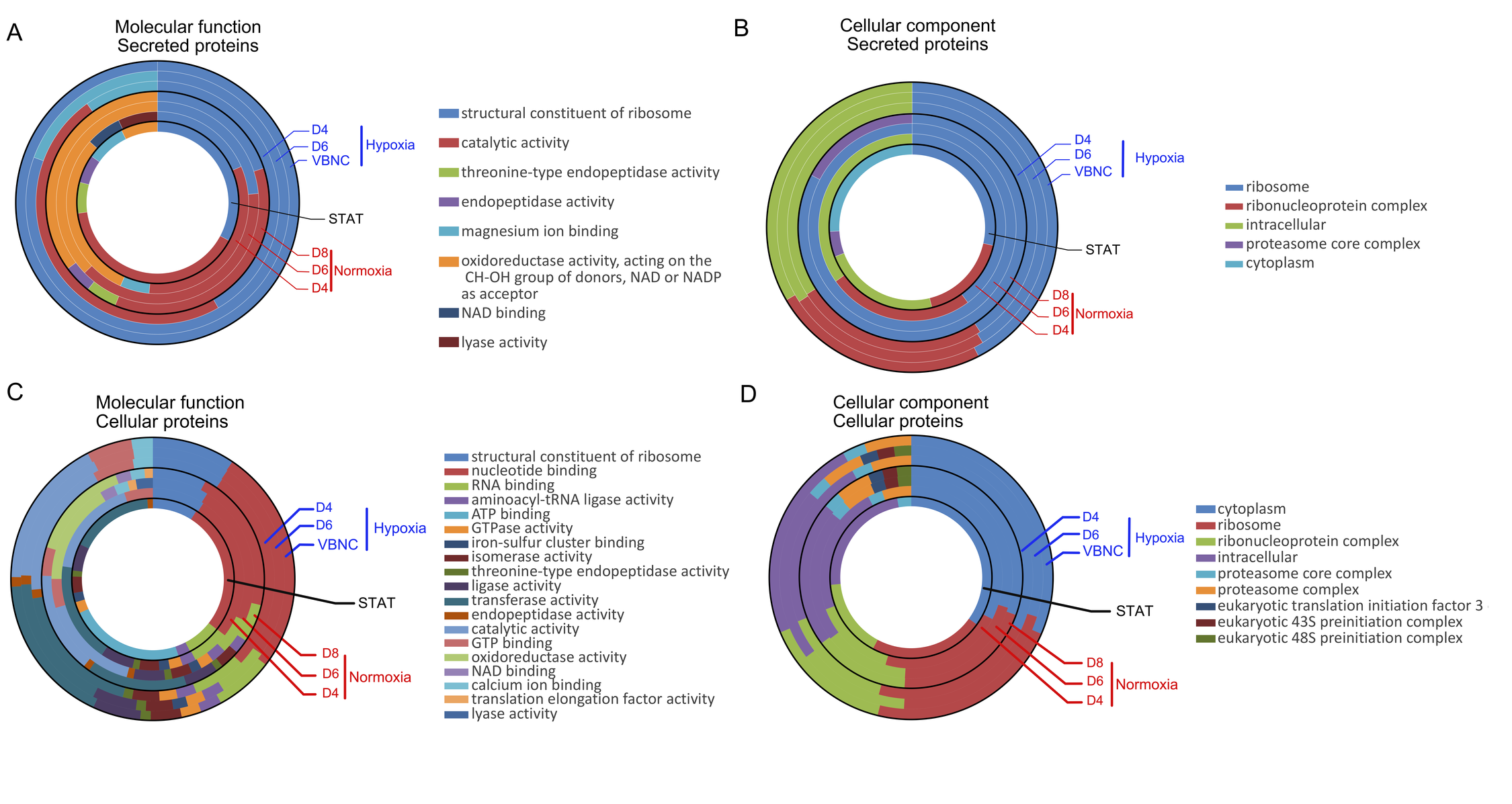

Supplement: S8 Fig — A. The major enriched molecular function process for secreted proteins in hypoxia was structural constituents of ribosome and in normoxia catalytic and oxidoreductase activity. B. For cellular component, the major enriched cellular components were the ribosome, ribonucleoprotein complex and intracellular in hypoxia and mainly ribosome in normoxia. C. The major enriched molecular function process for cellular proteins in hypoxia were the same as normoxia: nucleotide binding, transferase activity and catalytic activity. D. For cellular component, the major enriched cellular component for both hypoxia and normoxia were cytoplasm, ribosome, ribonucleoprotein complex and intracellular. (TIF) [file ppat.1007945.s017.tif]
